# The next generation of the World Health Organization's global antiretroviral guidance

**DOI:** 10.7448/IAS.16.1.18757

**Published:** 2013-06-30

**Authors:** Gottfried Hirnschall, Anthony D Harries, Philippa J Easterbrook, Meg C Doherty, Andrew Ball

**Affiliations:** 1 HIV Department, World Health Organization, Geneva, Switzerland; 2 International Union Against Tuberculosis and Lung Disease, Paris, France; 3 London School of Hygiene and Tropical Medicine, London, UK

**Keywords:** ARV guidelines, WHO, adults, pregnant women, adolescents, children

## Abstract

The 2013 World Health Organization’s (WHO) *Consolidated guidelines on the use of antiretroviral drugs for treating and preventing HIV infection* provide more than 50 new recommendations across the continuum of HIV care, including recommendations on HIV testing, using antiretroviral drugs for prevention, linking individuals to HIV care and treatment services, initiating and maintaining antiretroviral therapy (ART) and monitoring treatment. Guidance is provided across all age groups and populations of adults, pregnant and breastfeeding women, adolescents and key populations. The guidelines are based on a public health approach to expanding the use of ARV drugs for HIV treatment and prevention, with a particular focus on resource-limited settings.

The most important new clinical recommendations include: treating adults, adolescents and older children earlier – starting ART in all individuals with a CD4 cell count of 500 cells/mm^3^ or less (but giving priority to those with advanced clinical disease or a CD4 cell count less than 350 cells/mm^3^); starting ART at any CD4 cell count in certain populations, including those with active TB (existing recommendation), Hepatitis B infection and severe chronic liver disease, HIV-positive partners in serodiscordant couples (existing recommendation), pregnant and breastfeeding women, and children younger than 5 years of age; a preferred first-line ART regimen of Tenofovir+3TC or FTC+ Efavirenz as a once-daily fixed-dose combination for adults, pregnant women, and children aged 3 years and older; and the use of viral load testing as the preferred approach to monitoring the response to ART and to diagnose treatment failure. Guidance is also provided on enhancing the efficiency and effectiveness of HIV services, including strategies to improve retention in care, and adherence to ART; task-shifting to address human resource gaps; decentralizing delivery of ART to primary health care, and integrating ART services within maternal and child health, TB or drug dependency clinics. There is additional guidance for programme managers on how to plan HIV programmes and use resources most efficiently.

## Introduction and history

A core function of the World Health Organization (WHO) HIV programme is to translate new evidence, experience and technical innovations into global guidance to inform scale up of national antiretroviral (ARV) programmes. In 2002, WHO first published guidelines on the use of antiretroviral therapy (ART) among adults and adolescents [1], and in 2001 and 2004, on ARV use for the prevention of mother-to-child transmission (PMTCT) of HIV [2,3]. The 2006 and 2010 updates of these guidelines [4–9] used the concept of a public health approach, with simplified ART regimens, and in 2010, the CD4 threshold for ART initiation was raised from ≤200 cells/mm^3^ to ≤350 cells/mm^3^ [9]. This has facilitated impressive progress in the global scale up of ART. By the end of 2011, the majority of 80 low- and middle-income countries (LMICs) included in a survey had adopted the 2010 CD4 initiation threshold of less than 350 cells/mm^3^ in their national guidelines [10]. At the end of 2012, an estimated 9.7 million people in LMICs were receiving ART. This represents 65% of the global target of 15 million on ART by 2015 set by the UN General Assembly in 2011, and an increase of 1.6 million from the end of 2011 [11,12]. This increased ART access has been associated with declining HIV mortality and incidence rates in several countries. The global target of 15 million on ART by 2015 is now within reach [13], and a few countries (including resource-limited countries) have already reached or are close to achieving “universal access” to ART (defined as greater than 80% coverage of those eligible for ART).

However, several major challenges will need to be overcome if progress towards universal access is to be sustained. ART coverage is highly variable across countries, ages and populations [8,9]. In particular, the levels of ART coverage for children (younger than 15 years) and pregnant women eligible for ART for their own health are considerably lower than those for other adults [12]. Treatment access is also low in settings where the epidemic is concentrated among marginalized populations, such as sex workers, people who inject drugs, and men who have sex with men [12]. The majority of people living with HIV in LMICs are unaware of their HIV status [14]. In addition, late presentation is common, so ART is only initiated when disease is advanced and CD4 cell counts are well below the recommended threshold, resulting in a high risk of early mortality [12]. Finally, there is still a high attrition rate at all stages along the continuum of care [15].

## Why new consolidated guidelines?

Since the 2010 guidelines, a series of landmark studies have provided new evidence on the individual clinical and HIV prevention benefits of earlier ART [16–19]. This has led to new WHO guidance on its use for HIV prevention in serodiscordant couples [20], PMTCT [21], prevention of TB [22] and pre-exposure prophylaxis (PrEP) of HIV [23]. In addition, once-daily, fixed-dose combination ARV regimens for use in most populations and age groups have become more widely available and affordable in LMICs, and new testing approaches and technologies, including CD4 point-of-care assays, have enabled increasing decentralization of HIV testing and care.

As the HIV treatment and prevention benefits of ARVs become clearer, HIV programme managers are faced with a broadening array of options for their use in reducing HIV morbidity, mortality and transmission in different populations. Using ARVs most strategically requires careful decision making at the clinical, operational and programmatic levels. For example, changes are needed in service delivery models to support timely initiation of ART, optimal treatment adherence and retention in care across the continuum of care. In response to these advances, WHO has now updated and combined all its ARV-related guidance into one consolidated guidelines document: *Consolidated guidelines on the use of antiretroviral drugs for treating and preventing HIV infection* [24]. The primary target audience is national programme managers who make policy and planning decisions in settings with limited health system capacity and resources, but it also includes agencies that provide financial and technical support to HIV programmes, clinicians and other health service providers. The guidance addresses the clinical, operational and programmatic aspects of using ARV drugs for both HIV treatment and prevention across all age groups and populations, and across the entire continuum of care.

## What is different about the consolidated guidelines?

First, instead of separate ARV guidelines for adults (including pregnant women) and children, all age groups and populations are now covered, enabling a more harmonized approach to the choice of ARV regimens and treatment approaches. Second, the clinical guidelines adopt a continuum of care approach from diagnosis of HIV to the use of ARVs in prevention, pre-ART care, initiation and maintenance of first-, second- and third-line ART regimens, monitoring for treatment failure and ARV toxicity, management of co-infections and co-morbidities, and prevention and management of drug interactions. Third, in addition to the updated clinical recommendations, there is operational guidance on how to implement ARV programmes more efficiently and improve ART access, with recommendations on task shifting, decentralization, integration and adherence. Furthermore, there is programmatic guidance on how to support the translation of clinical and operational recommendations into policy and practice at national level, including what parameters to consider when setting priorities and deciding on the implementation of the recommendations. Fourth, new recommendations on the use of ARVs for treatment and prevention are harmonized with links to the existing WHO guidance on other major aspects of HIV diagnosis, ARV-related prevention, and HIV care and treatment. Finally, there is guidance on proposed indicators for monitoring implementation of new recommendations and programme performance across the continuum of care.

## The guidelines development process

The revision process for the 2013 guidelines was initiated in early 2012, and conducted in accordance with procedures established by the WHO Guidelines Review Committee [25]. Clinical and operational recommendations were developed using the GRADE system (Grading of Recommendations, Assessment, Development and Evaluation), which emphasizes a structured, explicit and transparent approach to rating the quality of evidence and strength of recommendations [26]. The decision-making process involved a critical review of the evidence based mainly on systematic reviews of randomized clinical trials and, where appropriate, observational studies. The process was further complemented by consideration and assessment of costs and resource implications, impact and cost-effectiveness modelling based on potential recommendations, feasibility of and barriers to implementation, surveys of community and health worker values and preferences, and equity and human rights implications. These were all considered to inform the weighing up of benefits and harms of potential recommendations.

As in the past, the implementation of the guidelines and scale up of ARV use are underpinned by several key guiding principles. The first principle is a public health approach that seeks to ensure the widest possible access to high-quality services at the population level, balancing the best evidence-based standard of care with the feasibility of large-scale implementation in resource-limited settings. Second is the core principle of human rights and health equity to guide national HIV policies and programmes, and so ensure that among those eligible for ART, priority is given to those most in need, and that care is provided in an environment that minimizes stigma and discrimination. The third guiding principle is optimizing the effectiveness and efficiency of HIV programmes across the continuum of care through a strategic mix of HIV testing approaches, improved adherence and retention measures, innovative service delivery models, harmonized drug regimens, and simpler and more affordable point-of-care diagnostics and laboratory services.

## Key changes in the 2013 guidelines

Table 1 summarizes the key clinical recommendations on when and what to start in the 2013 guidelines and how they differ from those in 2010.

**Table 1:** Comparison of key WHO recommendations on when to start ART and choice of drug regimen between 2010 and 2013 as well as operational recommendations

When to initiate ART (ARV-naive individuals)			

Population	Target population	2010 ART guidelines	2013 ART guidelines
Adults and adolescents*	HIV-infected individuals	CD4 cell count ≤350 cells/mm^3^ or WHO clinical stage 3 or 4 regardless of CD4 cell count	CD4 cell count ≤500 cells/mm^3^ or WHO clinical stage 3 or 4 regardless of CD4 cell count
	HIV-infected pregnant and breastfeeding women	CD4 cell count ≤350 cells/mm^3^ regardless of clinical symptoms or WHO clinical stage 3 or 4 regardless of CD4 cell count	Regardless of CD4 cell count or WHO clinical stage
	HIV-infected partners in serodiscordant couple relationship(s)	No recommendation established	Regardless of CD4 cell count or WHO clinical stage [20]
	HIV/TB co-infection	Presence of active TB disease, regardless of CD4 cell count	No change
	HIV/HBV co-infection	Evidence of chronic active HBV disease, regardless of CD4 cell count	Evidence of chronic HBV disease with severe liver disease (e.g., cirrhosis), regardless of CD4 cell count
Children	HIV-infected children ≥5 years old	CD4 cell count ≤350 cells/mm^3^ or WHO clinical stage 3 or 4 regardless of CD4 cell count	CD4 cell count ≤500 cells/mm^3^ or WHO clinical stage 3 or 4 regardless of CD4 cell count
	HIV-infected children 1–5 years old	1. Between 12 and 24 months of age, regardless of CD4 cell count or WHO clinical stage. 2. Between 24 and 59 months of age with CD4 cell count of ≤750 cells/mm^3^ or CD4% ≤25, or whichever is lower, regardless of WHO clinical stage.	Regardless of CD4 cell count and WHO clinical stage
	HIV-infected infants <1 year old	All infants, regardless of CD4 cell count and WHO clinical stage	No change

**What ART regimen to start with (ARV-naive individuals)**			

Adults and adolescents*	HIV-infected individuals HIV-infected pregnant and breastfeeding ARV-naive women HIV/TB co-infection HIV/HBV co-infection	AZT or TDF+3TC (or FTC)+EFV or NVP AZT+3TC+NVP or EFV AZT or TDF+3TC (or FTC)+EFV TDF+3TC (or FTC)+EFV	$\{\begin{array}{l} \begin{array}{l} \text{TDF + 3TC (or FTC)+EFV}\\ \text{(as a fixed-dose combination)} \end{array} \end{array}$
Adolescents* Children	Weighing <35 kg ≥3 years old <3 years old**	NVP or EFV+2 NRTI [AZT/3TC (preferred)] NVP+2 NRTI [AZT/3TC (preferred)]	ABC (or AZT or TDF)+3TC+EFV ABC (or AZT or TDF)+3TC+EFV ABC (or AZT)+3TC+LPV/r (regardless of PMTCT exposure)

* Adolescents are defined as aged 10 to 19 years inclusive.

** In children less than 2 years of age, previously exposed to NNRTIs, the use of a LPV/r based treatment was recommended.

### Earlier initiation of ART (CD4 cell counts<500 cells/mm^3^)

The new recommendations include expanding eligibility for ART initiation to a CD4 threshold of 500 cells/mm^3^ or less for adults, adolescents and children (five years and older), while prioritizing those in greatest need: those with advanced HIV disease or CD4 cell counts of 350 cells/mm^3^ or less. The recommendation for earlier ART initiation at a CD4 cell count between 350 and 500 cells/mm^3^ is supported by moderate-quality evidence of reduced morbidity and mortality from a systematic review of 21 observational studies and 3 randomized controlled trials [27], high-quality evidence of reduced HIV transmission from one randomized trial [16], and cost-effectiveness models that showed that expanding ART eligibility to ≤500 cells/mm^3^ may result in substantial health benefits and be cost effective in most epidemic settings [28].

In addition, no studies suggested individual-level harms from earlier ART initiation, although these studies were generally of limited duration. ART initiation regardless of CD4 cell count is also recommended for certain populations, namely pregnant women, HIV-positive partners in serodiscordant couples, children younger than five years of age, those with active TB disease, as well as HIV and hepatitis B virus co-infected individuals with evidence of severe chronic liver disease. There was insufficient evidence and/or favourable benefit–risk profiles to recommend initiating ART at CD4 cell counts over 500 cells/mm^3^ or regardless of CD4 cell count or WHO clinical stage in the following populations: in all adults and children above 5 years; key populations that are disproportionately affected by HIV (including men who have sex with men, transgender people, people who inject drugs, and sex workers); HIV-infected individuals who are 50 years of age and older; and those who are co-infected with HIV-2 or hepatitis C. The initiation of ART in these people should follow the same general principles and recommendations as for other adults and adolescents with HIV. Comprehensive WHO guidelines on management of hepatitis C will be published in 2013.

### Initiating life-long ART in pregnant women

In 2010, the WHO guidelines recommended life-long ART for pregnant women based on the 2010 eligibility criteria of a CD4 cell count of 350 cells/mm^3^ or less, and two complex prophylaxis options (Option A and Option B) for those not yet eligible for ART. Option A provided twice-daily zidovudine to the mother from 14 weeks of gestation to the onset of labour, and single-dose nevirapine and twice-daily zidovudine and lamivudine given for seven days post-partum. Option B provided triple ARV therapy to the mother until delivery or if breastfeeding was continued until one week after all infant exposure to breast milk had ended. Both prophylaxis options included four to six weeks of peripartum nevirapine prophylaxis given to the infants.

In 2013, in order to accelerate rapid global scale up of ART and PMTCT, to increase ART access for pregnant women, and to achieve the global goal of eliminating new paediatric infections, WHO recommends ART in one simplified regimen to all pregnant and breastfeeding women regardless of CD4 cell count during the period of risk of mother-to-child transmission (MTCT).

Continuation of life-long ART regardless of CD4 cell count or clinical status (Option B +) is recommended, particularly in generalized epidemic settings with high rates of fertility, and limited access to CD4 testing. Option B+ was first proposed and implemented in Malawi, and it provides important programmatic and clinical benefits [29], including harmonization of the ARV regimen with that used in non-pregnant adults, ease of implementation (women who are immune compromised can start ART straightaway without waiting for a CD4 cell count), and avoidance of stopping and starting drugs with repeat pregnancies and so provide early protection against MTCT in future pregnancies. Earlier ART may also reduce the high post-partum mortality, as well as offer protection from HIV transmission to the partner or spouse [16].

The 2013 recommendation is conditional, recognizing the current lack of conclusive evidence to support a universal ART strategy. Countries will have to weigh the benefits of continuing ARVs after the transmission risk is removed, and may opt for stopping ART after the breastfeeding period (Option B). Option A is no longer recommended.

### Harmonization of ART regimens across populations

The 2013 guidelines promote further simplification of ART delivery by recommending a once-daily fixed-dose combination of tenofovir, lamivudine or emtricitabine and efavirenz as the preferred first-line ART regimen for use across all populations of children (older than three years), adolescents, adults, pregnant women and women of reproductive ages. In children less than 3 years of age, a lopinavir-based regimen is recommended as first-line ART, regardless of NNRTI exposure. The recommendations for second-line regimens in 2013 remain unchanged since 2010, with heat-stable fixed-dose combinations, and atazanavir/ritonavir and lopinavir/ritonavir as the preferred boosted protease inhibitor (PI) options. However, WHO may include darunavir/ritonavir as an alternative PI option should there be a reduction in cost and a heat-stable fixed-dose combination tablet developed. The need to phase out stavudine in first-line ART regimens for adults and adolescents is further reinforced.

### Improved patient monitoring with viral load

Viral load testing is recommended as the preferred approach for monitoring ART response to provide an early and more accurate indication of treatment failure and more appropriate switching to second-line drugs, reducing the accumulation of drug-resistance mutations, and improving clinical outcomes. A pooled analysis showed the current WHO immunological and clinical criteria for treatment failure have poor sensitivity and positive predictive value for identifying virological failure in adults and children [30]. A systematic review of five randomized controlled trials and one observational study provides further support for virological monitoring [31].

### Task shifting, decentralization and integration

Treating many more people requires more efficient, acceptable and accessible health services. New operational recommendations emphasize decentralizing ART to primary health care services and integrating ART provision within TB, antenatal care, maternal and child health services, and drug-dependence services. The operational guidance also addresses the implications of new clinical recommendations for laboratory services and drug and diagnostics supply management.

## What are the key messages for HIV programme managers?

National programme managers play a unique role in managing the process of adapting and implementing the global recommendations for use at country level, and the programmatic guidance offers steps to ensure fair, inclusive and transparent decision-making processes. In all settings, the priority should be to treat those people who are in greatest need of ART for their own health. Further expansion of ART access and the trajectory towards the implementation of other recommendations may involve various policy combinations depending on the local context. Factors to be considered include HIV epidemiology, current ART coverage, availability of resources and anticipated cost effectiveness, and the capacity of the health system. The aim is to achieve full implementation of the recommendations as rapidly and efficiently as possible, ensuring quality, sustainability and maximum impact of programmes.

Ultimately, the process of guideline revision and adaptation is the responsibility of national stakeholders. Different approaches may be necessary and equally valid depending on the context. Each country will have to plan its own approach to ensure that the current ARV programmes are scaled up and sustained, and that expanded access is fair and equitable. Finally, countries will also have to align their HIV priorities with their broader health and development strategies.

## What are the country-level implications of these guidelines?

If treatment scale up is to be sustained, this will require further reductions in ARV drug costs, as well as continued efficiencies and innovations in service delivery. These include simplified HIV diagnostic and laboratory processes (point-of-care and dried blood spot technologies, low-cost rapid tests, and well-planned supply chain management), linking decentralized approaches with task shifting, and requisite training and supervision of health staff. While such efforts to increase efficiency and reduce costs are important, a balance will have to be struck so that quality is not compromised. Improving long-term patient retention and treatment adherence through strengthened linkages along the continuum of care, better ARV formulations (especially for infants and young children) and future strategies (sustained-release once-a-week/month regimens), and a reliable supply chain for drugs and diagnostics will also be critical for limiting the further spread of drug resistance. Finally, as HIV increasingly becomes a chronic manageable condition and people with HIV develop non-communicable diseases (NCDs) related to HIV itself, ageing or ARV complications, closer links between HIV services and other NCD health services become increasingly important.

## What are the required global investments and estimated impact?

Estimates in 2011 indicated that an effective global HIV response (including treatment provision based on the 2010 guidelines) would cost US$22–24 billion annually in 2015 [32]. If fully implemented, the 2013 guidelines would increase the number of people eligible for ART globally to 25.8 million (compared with 16.7 million under the 2010 guidelines). Assuming that ART coverage increases gradually to around 80% of the total number of people eligible for ART, the estimated annual cost of reaching these numbers in 2025 would be an increase of about 10%, or US$2–4 billion per year (in addition to the current US$22–24 billion for a comprehensive HIV response). It is projected that the resource needs would level off over time and even start to decline after 2025, reflecting the accumulated prevention benefits of expanded ART provision. If the recommendations are fully implemented, it is projected that by 2025, the annual mortality would fall from 1.3 to 0.8 million, and 3.5 million additional new HIV infections would be prevented [12]. Changing the eligibility criteria is rated as cost effective, with the cost per quality-adjusted life year saved less than US$600 and less than US$5000 per new HIV infection averted.

Estimating impact and costs associated with the implementation of new recommendations is a key step in the roll-out process at country level. As the impact of changes in the eligibility criteria will differ from country to country, several online costing tools and resources (Spectrum and OneHealth [33]) have been developed to help countries model the impact that increasing ART access is likely to have on mortality, HIV incidence as well as associated costs, and are highlighted in the programmatic guidance.

## What comes next?

Implementing the 2013 guidelines represents an important step towards the goal of achieving universal access to HIV prevention and treatment [12], as well as increasing the efficiency, impact and long-term sustainability of ARV programmes. In the longer term, the guidelines will also contribute to and inform efforts to achieve universal health coverage, a key pillar of the post-2015 development agenda.

The 2013 guidelines process also identified key gaps in knowledge and priority areas for operational and implementation research and for inclusion in the 2015 update. These include: evaluating the impact of earlier ART initiation on patient acceptability, adherence, retention, incidence of toxicities and treatment failure in different populations, especially pregnant women; the impact of the new service delivery models of integration and decentralization; establishing optimal short- and long-term approaches for monitoring ART response and toxicities; and management of key co-morbidities, including NCDs.

HIV treatment and care continues to evolve rapidly, and the guidelines will be comprehensively reviewed and updated regularly as new evidence emerges and practice evolves in the use of ARV drugs. When necessary, rapid guidance and technical and programmatic updates will complement the biannual updates. WHO headquarters will work closely with regional and country offices and implementing partners to ensure wide dissemination of the updates through regional and sub-regional meetings.
